# LncRNA KCNA2-AS regulates spinal astrocyte activation through STAT3 to affect postherpetic neuralgia

**DOI:** 10.1186/s10020-020-00232-9

**Published:** 2020-11-23

**Authors:** Cunlong Kong, Jie Du, Huilian Bu, Chen Huang, Fuxing Xu, Huan Ren

**Affiliations:** 1 Center of Pain Management, Department of Anesthesiology, Pain and Perioperative Medicine, The First Affiliated Hospital of Zhengzhou University, Zhengzhou, 450052 China; 2 Outpatient and Emergency Department of West District Hospital, Children’s Hospital Affiliated to Zhengzhou University, Zhengzhou, 450018 China

**Keywords:** Postherpetic neuralgia, Spinal astrocytes, LncRNA KCNA2-AS, pSTAT3, GFAP

## Abstract

**Objectives:**

Postherpetic neuralgia (PHN) is the most common complication of herpes zoster, but the mechanism of PHN is still unclear. Activation of spinal astrocytes is involved in PHN. Our study aims to explore whether lncRNA KCNA2 antisense RNA (KCNA2-AS) regulates spinal astrocytes in PHN through signal transducers and activators of transcription 3 (STAT3).

**Methods:**

Varicella zoster virus (VZV)-infected CV-1 cells were injected into rats to construct a PHN model. Primary spinal cord astrocytes were activated using S-Nitrosoglutathione (GSNO). Glial fibrillary acidic protein (GFAP; marker of astrocyte activation), phosphorylated STAT3 (pSTAT3), and KCNA2-AS were analyzed by immunofluorescence and RNA fluorescence in situ hybridization. RNA pull-down and RNA immunoprecipitation were used to detect binding of KCNA2-AS to pSTAT3.

**Results:**

KCNA2-AS was highly expressed in the spinal cord tissue of PHN model rats, and was positively correlated with GFAP expression. GFAP was significantly increased in GSNO-induced cells, but the knockdown of KCNA2-AS reversed this result. Meanwhile, pSTAT3 was significantly increased in GSNO-induced cells, but knockdown of KCNA2-AS reduced pSTAT3 within the nucleus while the total pSTAT3 did not change significantly. pSTAT3 bound to KCNA2-AS and this binding increased with GSNO treatment. Furthermore, knockdown of KCNA2-AS in PHN model rats relieved mechanical allodynia.

**Conclusion:**

Down-regulation of KCNA2-AS alleviates PHN partly by reducing the translocation of pSTAT3 cytoplasm to the nucleus and then inhibiting the activation of spinal astrocytes.

## Introduction

Herpes zoster is an acute infectious skin disease caused by varicella zoster virus (VZV). Postherpetic neuralgia (PHN) is the most common complication of herpes zoster, which occurs in about one in five patients and severely affecting their quality of life (Saguil 2017). The prevalence of postherpetic neuralgia increases with age, and 80% of patients are over 50 years old (Yawn and Gilden 2013). Nevertheless, about one-third of patients with PHN do not respond to routine treatment (Gossrau 2014). Therefore, it is necessary to explore new treatment strategies.

Astrocytes account for about 20–40% of all glial cells in the central nervous system, and their main role is to provide structural and nutritional support for neurons (Herculano-Houzel 2014). The activation of astrocytes has been shown to be a key driver of neuropathic pain (Ji et al. 2019; Sommer et al. 2018; Hansen and Malcangio 2013). Moreover, some studies have shown that spinal astrocytes were obviously activated in the PHN model, and the injection of astrocyte-specific inhibitors could obviously alleviate the mechanical allodynia and spinal central sensitization (Zhang 2011; Zhang et al. 2009). Therefore, inhibiting the activation of spinal astrocytes may be a new therapeutic strategy for PHN.

Transcription factor signal transducers and activators of transcription 3 (STAT3) has been shown to promote proliferation and activation of astrocytes in the spinal cord after nerve injury (Tsuda 2011; Wu et al. 2020). Meanwhile, inhibiting the activation of the STAT3 signaling pathway can also relieve neuropathic pain (Ge 2018; Wang 2018). Furthermore, blocking the STAT3 pathway in S-Nitrosoglutathione (GSNO; NO donor)-activated astrocytes can inhibit the expression of glial fibrillary acidic protein (GFAP; a marker of astrocyte activation (Eng and Ghirnikar 1994; Chen 2018; Brahmachari et al. 2006), indicating that STAT3 pathway is involved in GFAP production in NO-activated astrocytes.

Long non-coding RNA (lncRNA) plays a crucial role in various life activities of the organism and is involved in the occurrence and progression of multiple diseases (Jarroux et al. 2017; Beermann 2016). Although lncRNAs are rarely reported in PHN, numerous studies have shown that lncRNAs are involved in the progression of neuropathic pain (Li 2019; Wu et al. 2019). For example, overexpression of lncRNA KCNA2 antisense RNA (KCNA2-AS) inhibits potassium voltage-gated channel subfamily A member 2 (KCNA2) to increase the excitability of DRG neurons and neuropathic pain symptoms (Li 2019); inhibition of NEAT1 can alleviate neuropathic pain in CCI rats by regulating miR-381/HMGB1 axis (Xia et al. 2018). In addition, several studies claimed that lncRNAs were involved in regulating the activation of astrocytes. For instance, upregulation of the lncRNA MEG3 inhibited activation of astrocytes in hippocampus tissues in Alzheimer's disease (Yi 2019); knockdown of lncRNA PVT1 could inhibit the activation of astrocytes in hippocampus tissues of rats (Zhao et al. 2019). These results suggest that lncRNAs may also play an important role in astrocyte activation during the pathological process of PHN.

We found that the level of KCNA2-AS changed significantly in the PHN model, but its specific mechanism of action is unclear. This study investigated whether KCNA2-AS is involved in the pathological process of PHN and explored the underlying mechanism.

## Materials and methods

### Animals

Sprague-Dawley (SD) rats (Female, 7–8 weeks old) were purchased from the experimental animal center of the Zhengzhou University, and were kept in light/dark cycle for 12 h at 22–25 °C with free feeding. The animal experimental procedures were approved by the Animal Care Committee of the First Affiliated Hospital of Zhengzhou University and performed in accordance with the National Institutes of Health Guide for the Care and Use of Laboratory Animals. According to the method in literature (Dalziel 2004), the postherpetic neuralgia (PHN) model was established by infecting SD rats with VZV. African green monkey kidney fibroblast CV-1 cells were purchased from ATCC (Gaithersburg, MD, USA), and grown and maintained in Dulbecco’s modified Eagle’s medium/F12 (DMEM/F12; Gibco, Grand Island, New York, USA) supplemented with 10% foetal calf serum (FBS; Gibco). First, DMEM/F12 was used to make CV-1 cells into a cell suspension and seeded in a culture flask (5 × 10^4^ cells/cm^2^). CV-1 cells were then infected with VZV (ATCC). When about 80% of the cells exhibited cytopathic effect (cells aggregate into large clumps or nuclei swell and rupture), the cells were collected with PBS (Gibco) and serum from SD rats was added to avoid hypersensitivity during the subcutaneous injection. Finally, 50 μl cell suspension (about 5 × 10^6^ cells infected cells) were injected into the sole of the right hind limb (PHN group). Control rats were injected with 50 μl uninfected CV-1 cells (Sham group) and fed separately from PHN group rats. 20 rats in each group, 5 rats were sacrificed at random every other week after injection, and spinal cord tissues around L4, L5 were collected.

In the later experiment, SD rats were infected with LV-si-KCNA2-AS. Construction of lentiviral vectors, screening of si-KCNA2-AS, and packaging and purification of lentiviral vectors were all synthesized by GenePharma (Shanghai, China). After the rats were anesthetized, and the lentiviral Lv-si-KCNA2-AS (MOI = 100) was withdrawn into a 10 μl syringe, and the needle was inserted between the L4 and L5 vertebrae. When the rat flicked, the virus was injected into the spinal cavity (PHN + si-KCNA2-AS group). Rats in the control group were injected with saline of the same volume (PHN group). Three days later, the PHN model is constructed in the same way as above. Rats were sacrificed after 2 weeks and spinal cord tissues around L4, L5 were collected. qRT-PCR, Western Blot, and Immunofluorescence were executed (n = 4).

### Paw withdrawal threshold (PWT)

To assess mechanical allodynia, rats were tested for PWT. Rats were placed in plastic partitions individually with a metal mesh grid floor (5 mm × 5 mm), adapted to the environment until the sniffing reaction of rats were disappeared. The plantar surface of rat hind paw was subjected to pressure created by the calibrated Electronicvon Frey filament and the pressure gradually increased. When the rat showed a foot withdrawal reaction, or when the rat licked the toes detected, the test was stopped and the value was recorded. Each test should be measured at least 90 s apart for 5 consecutive times (Jia, et al. 2020).

### Examination of nitric oxide (NO) content

The nitric oxide (NO) assay kit (Nanjing Jiancheng, Nanjing, China) was used to determine the level of NO in rat spinal cord tissue. The rat spinal cord tissues were prepared into a homogenate, and the supernates were collected by centrifugation. 0.5 ml of tissue supernate was added with 0.4 ml of mixed reagent and placed in a 37 °C water bath for 60 min. 0.2 ml of reagent three and 0.1 ml of reagent four are added and mixed for 30 s. After 40 min at room temperature, the above samples were centrifuged at 4000 rpm for 10 min, and then 0.8 ml of supernate was added with 0.6 ml developer. After 10 min at room temperature, the absorbance was measured at a wavelength of 550 nm. The blank group and standard group were established according to the instructions.

### Primary rat spinal cord astrocytes

As mentioned above, primary spinal astrocytes were isolated from postnatal day 1–2 SD rats (Kerstetter and Miller 2012; Sun 2017). Briefly, the rats were anesthetized and decapitated. The spinal cord is then separated and cut into small pieces, and treated with 0.25% trypsin (Gibco). The supernate was passed through sterile nylon sieves and placed into DMEM (Gibco) containing 10% fetal bovine serum (FBS; Gibco). The cells were maintained in a humidified incubator with 95% air and 5% CO_2_ atmosphere at 37 °C. The culture medium was changed after 24 h. After cultured for 4–5 days, flasks were shaken at 200 rpm overnight at 37 °C to remove microglia and oligodendrocytes.

Primary rat spinal cord astrocytes were treated with 200 μM s-nitrosylglutathione (GSNO; Santa cruz biotechnology, Santa cruz, CA, USA) for different time (2, 6,12 and 24 h). Cells are then collected and tested.

### Cell transfection

Si-KCNA2-AS was synthesized by GenePharma (Shanghai, China). Lipofectamine 2000 (Invitrogen) was used to transfect primary rat spinal cord astrocytes. Cells were seeded in a 24-well plate and transfected when the cells reached more than 85%. 50 μl serum-free Opti-MEM I (Gibco) diluted 0.8 μg si-KCNA2-AS. Meanwhile, 50 μl serum-free Opti-MEM I diluted 2 μl Lipofectamine™ 2000. Then the two solutions were mixed and allowed to stand for 5 min. 100 μl of the mixed solution was added to each well, and the medium was refreshed after 12 h. The cells were treated with GSNO (200 μM; Santa cruz biotechnology) after transfection for 24 h.

### Quantitative real-time PCR (qRT-PCR)

The total RNA of spinal cord tissues or primary rat spinal cord astrocytes were extracted by RNA simple Total RNA Extraction Kit (Tiangen, Beijing, China). Then, the RNA was then reverse-transcribed into cDNA using the InRcute lncRNA cDNA Synthesis Kit (Tiangen). qRT-PCR was performed on the ABI 7500 Real-Time PCR system (Applied Biosystems, Carlsbad, USA) using SYBR Premix Ex Taq (TaKaRa, Dalian, China) and calculated by the 2^−ΔΔCt^ method. The U6 and GAPDH were used as the normalized internal reference. The sequence information of primers was showed in Table 1.

**Table 1:** Primers used for qRT-PCR

Genes	Forward (5′–3′)	Reverse (5′–3′)
KCNA2-AS	CTGAGGACAGCCAGGAGGA	GCTTGAGGGACAGTGAGATG
H19	GATGGAGAGGACAGAAGGACAGT	GAGAGCAGCAGAGATGTGTTAGC
uc-48+	GTTGGCAGTTCTGCAAGTA	GTTGGCAGTTCTGCAAGTAG
BC168687	CACCACCTGGATGACATGCT	GGTGGCATCCTTTGACTGGA
XIST	CGGGTCTCTTCAAGGACATTTAGCC	GCACCAATACAGAGGAATGGAGGG
GFAP	ATGGAGCGGAGACTGATCACC	GCGGAATGGTACCCAGGTGTC
GAPDH	GCTGGTGCCGAGTATGTT	CAGAAGGTGCGGAGATGA

### Western Blot (WB)

Spinal cord tissues or primary rat spinal cord astrocytes were lysed with RIPA lysis buffer (Biyuntian, Shanghai, China) containing protease and phosphorylation inhibitors (Biyuntian). The concentration of the protein was measured by a BCA kit (Biyuntian) according to the instruction. These proteins were subjected to sodium dodecyl sulfate-polyacrylamide gel electrophoresis (SDS-PAGE), and then transferred onto the polyvinylidene fluoride (PVDF) membranes (Millipore, Bedford, USA). The PVDF membrane was incubated in the closed buffer solution and incubated overnight with anti-GFAP antibody (ab7260, abcam, 1:10,000), anti-GAPDH antibody (ab9485, abcam, 1:2000), anti-STAT3 antibody (ab68153, abcam, 1:1000), anti-pSTAT3 antibody (ab76315, abcam, 1:5000) or anti-Lamin B1 antibody (ab16048, abcam, 0.1 µg/ml) at 4 °C overnight. After that, the second antibody (Biyuntian, 1:2000) was used for incubation for 4 h at 4 °C. The target proteins were evaluated with the chemiluminescence (ECL) system (Beyotime). GAPDH was used as a control for the total protein amount, and Lamin B1 was used as a control for the nucleus protein amount.

### Isolation of cytoplasmic and nuclear fractions from cells

Cytoplasmic and nuclear RNA or protein was extracted from primary rat spinal cord astrocytes by the PARIS™ Kit (Thermo Scientific, Waltham, MA, USA) according to the manufacturer’s instructions. About 1 × 10^6^ cells were collected and ice-cold Cell Fractionation Buffer was added. The nuclear lysate was gently mixed and incubated on the ice for 10 min and centrifuged at 4 °C for 500*g* for 5 min. The supernate was the cytoplasm and the precipitation was the nucleus. RNA isolation: the nuclear lysate was mixed with an equal volume of 2× Lysis/Binding Solution; then, an equal volume of 100% ethanol was added, and the sample mixture was passed through a Filter Cartridge by centrifugation; the Filter Cartridge was washed with the Wash Solution, then Elution Solution was used to eluate the RNA, and stored at − 80 °C. Protein isolation: nuclear lysates were incubated on ice 10 min, and the viscosity was reduced by sonicating the lysate; cytoplasmic lysates did not require another incubation on ice. The resulting RNAs were analyzed by qRT-PCR, and the resulting proteins were measured by Western blot.

### Immunofluorescence

Cells were rinsed with cold PBS (Gibco), and they were then fixed in 4% paraformaldehyde (Solarbio, Beijing, China) for 20 min. The cells were incubated in 1% BSA/5% serum/0.3 M glycine in 0.1% PBS-Tween for 1 h at room temperature, then incubated with anti-GFAP antibody (ab7260, abcam) or anti-pSTAT3 antibody (ab76315, abcam) overnight at 4 °C. Then, they were incubated with Goat Anti-rabbit IgG H&L (Alexa Fluor^®^ 647, ab150167; abcam) for 1 h at the room temperature shielded from light. 4′,6-diamidino-2-phenylindole (DAPI; Sigma-Aldrich, St. Louis, Missouri, USA) was used for nuclear staining at 37 °C for 2 min, and the cells were then washed three times with PBS and observed by using an inverted fluorescence microscope (Olympus Optical, Ltd., Tokyo, Japan).

The spinal cord tissues of rats sacrificed at 1 w, 2 w and 4 w in PHN group and sham group were taken and washed three times with PBS. 10% normal serum was added and sealed at 37 °C for 45 min. Then, anti-GFAP antibody (ab7260, abcam) and anti-iNOS antibody (ab15323, abcam) were incubated overnight at 4 °C. Next, they were incubated with Goat Anti-rat IgG H&L (Alexa Fluor^®^ 647, ab150167; abcam) and Goat Anti-Rat IgG H&L (Alexa Fluor^®^ 488, ab150165; abcam) for 1 h at the room temperature in the dark, DAPI (Sigma-Aldrich) was used for nuclear staining at 37 °C for 15 min, and then they were washed three times with PBS and observed by using an inverted fluorescence microscope (Olympus Optical).

### RNA fluorescence in situ hybridization (FISH)

The cells were fixed in 4% paraformaldehyde (Solarbio) for 15 min at room temperature, and then incubated with 0.2% Triton X-100 for 10 min. Fluorescence-conjugated KCNA2-AS probe (Sangon, Shanghai, China) was used to perform hybridization in the dark overnight. The FISH Tag™ RNA Multicolor Kit (Thermo Scientific) was used for the experiment. The hybridization buffer was prepared followed the manufacturer's guidelines. After the cells were treated with proteinase K, the hybridization buffer was added at 55 °C for 1 h. The probe was incubated in an 80 °C water bath for 2 min and placed on ice for 5 min, and then dissolved in hybridization buffer. The hybridization buffer was removed from the samples, the hybridization buffer containing the probe was added, and the mixture was kept at 55 °C from light overnight. Then, the mixture was washed 3 times with PBT solution and added DAPI (Sigma-Aldrich) for staining. Fluorescence images were obtained using an inverted fluorescence microscope (Olympus Optical).

### RNA pull-down assay

The biotinylated DNA probe complementary to KCNA2-AS was synthesized (Genepharma), and NC was a biotin-labeled antisense KCNA2 (Genepharm). Biotinylated KCNA2-AS or NC was purified and then was transfected into primary rat spinal cord astrocytes. Cells were harvested after 48 h using Lysis Buffer (Thermo Scientific). RNA pull-down assays were performed by using an RNA Pull-Down Kit (Thermo Scientific). Streptavidin Magnetic Beads were washed with 0.1 M NaOH and 50 mM NaCl twice, and were washed in 100 mM NaCl once. Following the instructions to configure the mixture to label the target RNA 50 pmol of RNA which was dropped in the precipitates, then 50 µl streptavidin magnetic beads were mixed in tubes and put into a magnetic stand to discard the supernatant. After washing with 20 mM Tris for three times, the beads were resuspended with an equal volume of 1× RNA Capture Buffer. 50 pmol of labeled RNA was added to the beads and incubated for 30 min at room temperature with agitation. The tubes were put in a magnetic stand to collect the beads, and an equal volume of 20 mM Tris washed the beads. Then, 100 µl of 1× Protein-RNA Binding Buffer was added to the beads. The supernatant was separated using a magnetic stand and washed three times with an equal volume of 1× wash buffer. 50 µl of Elution Buffer was added to the beads and mixed well by vortexing, and incubated 15–30 min at 37 °C with agitation. The samples were added reducing sample buffer to 1×. The eluted samples were heated for 5–10 min at 95–100 °C. The level of pSTAT3 in the complex was detected by Western Blot.

### RNA immunoprecipitation (RIP)

Primary rat spinal cord astrocytes were lysed with RIPA lysis buffer (Beyotime) and centrifuged. RIP assay was executed using the Magna RIP RNA-Binding Protein Immunoprecipitation Kit (Millipore, USA). The RIP Immunoprecipitation Buffer and magnetic beads were prepared according to the instructions. The magnetic beads were added to 900 µl of RIP Immunoprecipitation Buffer to each tube. The lysates were centrifuged at 14,000 rpm at 4 °C for 10 min, and 100 µl supernatant was added to each tube. Meanwhile, 10 µl the supernatant of lysate as the input. All the tubes were added to anti-pSTAT3 (ab76315, abcam) or IgG (Millipore), and incubated with rotating for overnight at 4 °C. The immunoprecipitation tubes were briefly centrifuged and placed on a magnetic separator and discarded the supernatant. Then, the magnetic beads were washed 5 times with 0.5 ml RIP Wash Buffer and 0.5 ml cold RIP Wash Buffer, respectively. Finally, RNA was purified with proteinase K buffer, and RNA was collected with 20 µl RNase-free water. The coprecipitated KCNA2-AS was detected by qRT-PCR.

### Statistical analysis

Statistics were analyzed by SPSS 22.0, and all data were presented as mean ± standard deviation (SD). The difference between two groups were compared by using the Student's *t*-test. The difference among multiple groups was compared by using the one-way analysis of variance (ANOVA) followed by the LSD post hoc test. *P* value < 0.05 was considered statistically significant.

## Results

### KCNA2-AS was highly expressed in the spinal cord of PHN model rats, and was correlated with GFAP expression

**Fig. 1:**
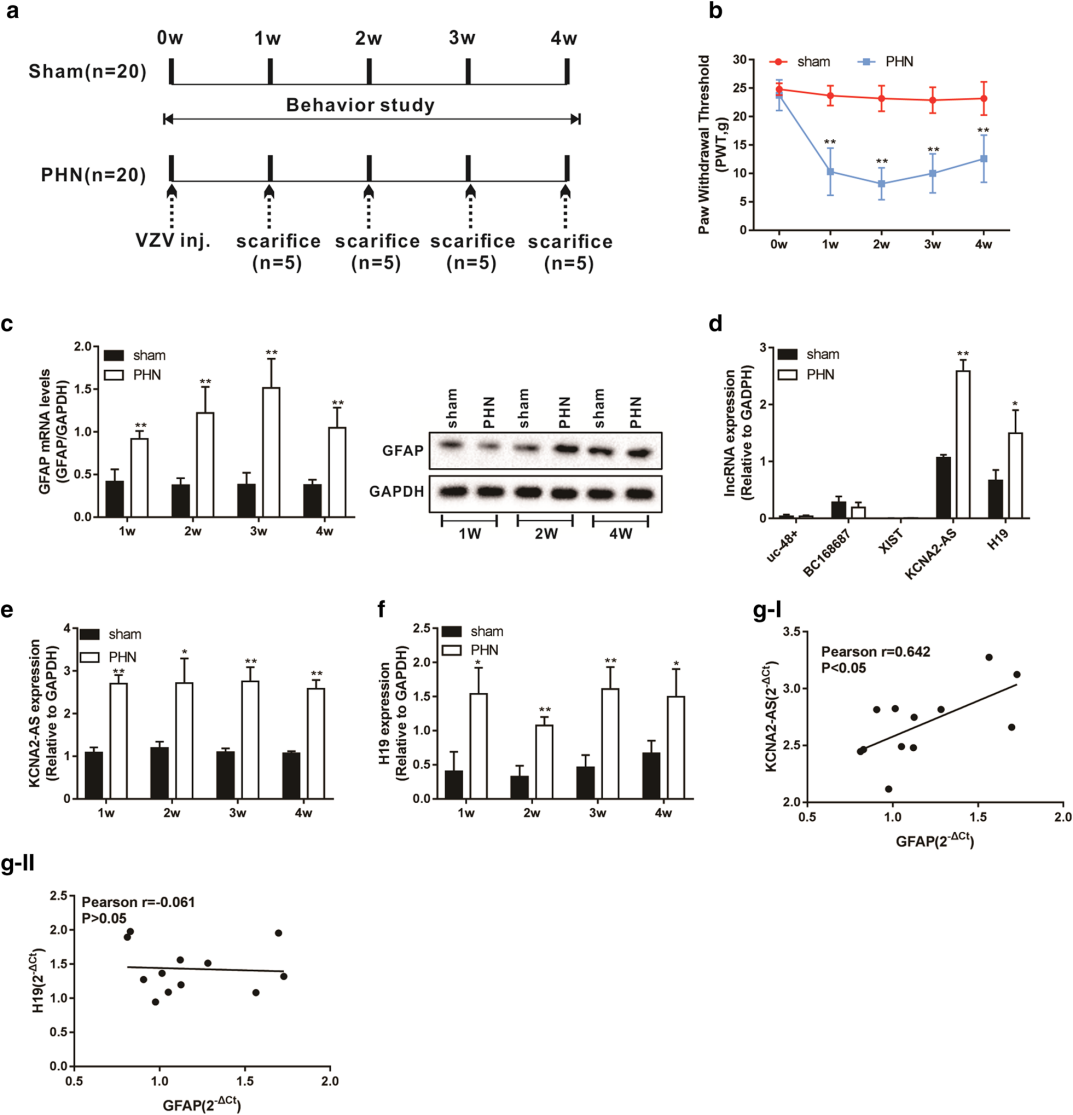
KCNA2-AS was highly expressed in the spinal cord of PHN model rats, and was correlated with GFAP expression. Varicella zoster virus-infected CV-1 cells (monkey kidney cell line), and 5 × 10^6^ cells were injected into rats (PHN group) to construct a PHN rat model. Sham group rats were injected with uninfected CV-1 cells. The experiment lasted 4 weeks, and 5 rats were sacrificed in each group every other week. **a** Schematic diagram of rat treatment. **b** Mechanical allodynia was detected by (PWT) before injection or sacrifice in rats. **c** The mRNA and protein levels of GFAP were detected by qRT-PCR and Western blot. **d** The expression of five lncRNAs in the spinal cord tissue of rats sacrificed at week 4 was detected by qRT-PCR. **e**, **f** The expression levels of KCNA2-AS and H19 were detected by qRT-PCR at different time points. **g** The correlation between the expression of KCNA2-AS or H19 and GFAP mRNA were analyzed. *P < 0.05 and **P < 0.01 vs. sham group

We injected VZV-infected CV-1 cells into rats to construct a PHN rat model, rats were injected with uninfected CV-1 cells as the control group (sham). These rats were sacrificed at different times (Fig. 1a). Mechanical allodynia was detected by PWT before injection or sacrifice in rats. The results showed increased mechanical allodynia compared with the sham group (Fig. 1b), indicating the successful establishment of the PHN model in rats. We detected the mRNA and protein levels of GFAP [a marker of astrocyte activation (Eng and Ghirnikar 1994; Chen 2018)] in the spinal cord tissue, and the results showed that the levels of GFAP mRNA and protein in the PHN group were significantly elevated compared with the sham group (Fig. 1c).

We selected five lncRNAs (uc-48+, BC168687, XIST, KCNA2-AS, and H19) that are involved in neuropathic pain to investigate whether they are involved in PHN (Li 2019; Wu et al. 2019). The expressions of five lncRNAs in the spinal cord tissue of rats sacrificed at week 4 were detected by qRT-PCR. Our results showed that the level of KCNA2-AS and H19 were significantly elevated in the PHN group compared with the sham group (Fig. 1d). To further detect the expression of KCNA2-AS and H19 in the PHN model, we detected the level of KCNA2-AS and H19 in spinal cord tissues at different time points. We found that the expression levels of KCNA2-AS and H19 at different time points were significantly increased, compared with the sham group (Fig. 1e, f). In addition, we analyzed the correlation between the expression of KCNA2-AS or H19 and GFAP mRNA, and the results showed that KCNA2-AS was positively correlated with GFAP (Fig. 1g). To explore the relationship between lncRNA and the activation of spinal astrocytes in PHN, we chose KCNA2-AS for further research.

### NO expression was increased in the spinal cord of PHN model rats

**Fig. 2:**
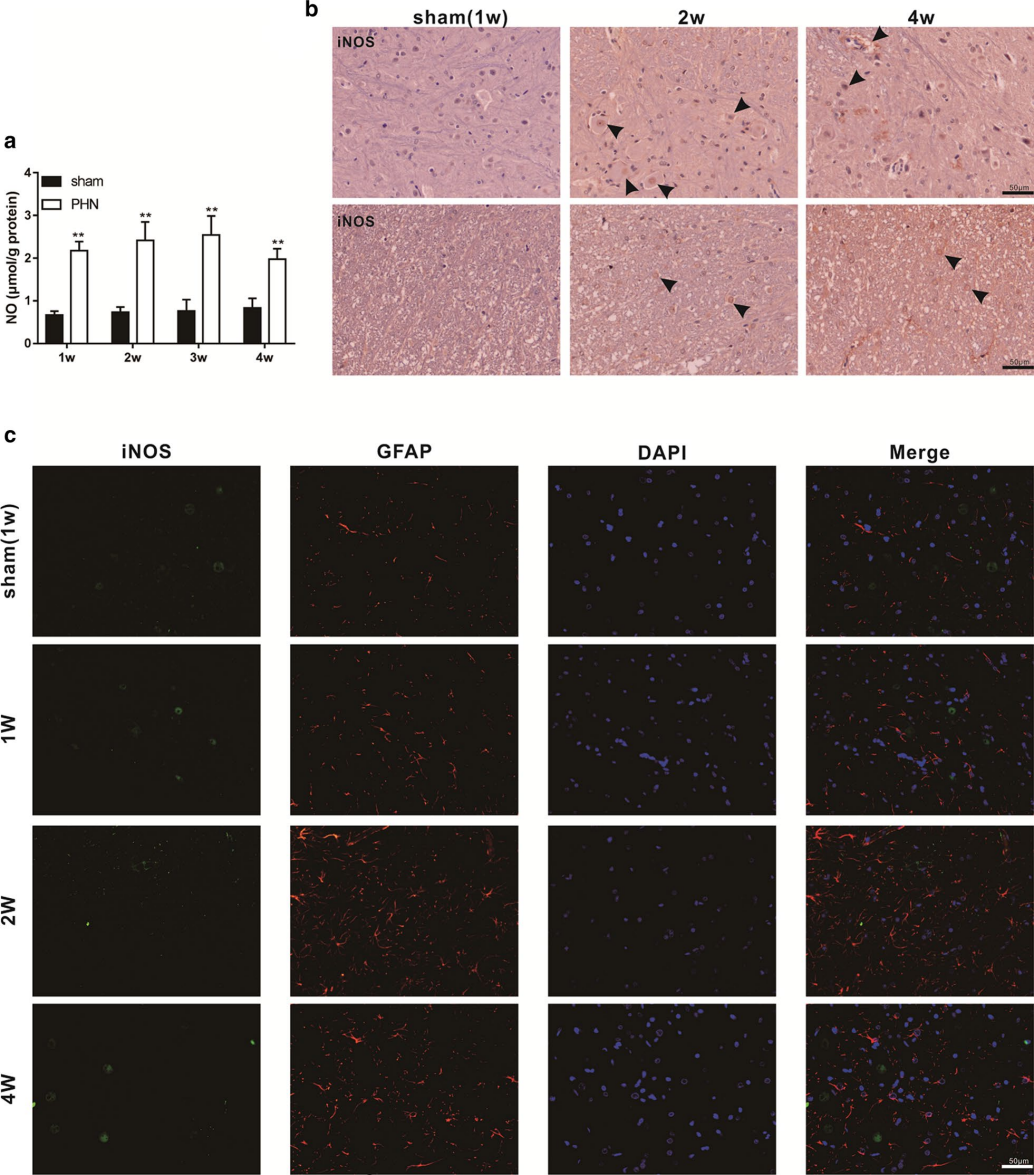
NO expression was increased in the spinal cord of PHN model rats. The PHN rat model was constructed with a consistent approach to the above description. The sham group rats were injected with uninfected CV-1 cells as control. The experiment lasted 4 weeks, and 5 rats were sacrificed in each group every other week. **a** The level of nitric oxide (NO) was detected. **b** Spinal cord tissues were collected from the PHN group and sham group at 1 w, 2 w and 4 w, immunohistochemical staining was used to detect the level of NO. **c** Spinal cord tissues were collected from the PHN group and sham group at 1 w, 2 w, and 4 w, respectively, and immunofluorescence staining was performed by using NOS/GFAP. **P < 0.01 vs. sham group

Nitric oxide (NO) has been shown to mediate the activation of astrocytes in the spinal cord and to promote neuropathic pain (Zhang 2011; Schmidtko et al. 2009). Our results showed that the level of NO in the spinal cord homogenate was observably elevated in the PHN group compared with the sham group (Fig. 2a). These data suggest that astrocytes were activated in the spinal cord of PHN model rats. The iNOS is an inducible isoform of NO and controls the production of NO. We performed the immunohistochemical staining and found that the level of iNOS was elevated in PHN group compared with the sham group, and the iNOS was mainly produced by neurons showed in the area that the arrows points to (Fig. 2b). Spinal cord tissues of rats sacrificed at 1 w, 2 w, and 4 w in the PHN group and sham group were co-stained with iNOS/GFAP immunofluorescence. The results showed that the number of GFAP positive cells was increased in the spinal cord of PNH model rats, but astrocytes were not the main cells that produced iNOS (Fig. 2c), implying that the astrocytes were dominating affected by the exogenous NO.

### The suppression of KCNA2-AS inhibits the activation of NO to astrocytes

**Fig. 3:**
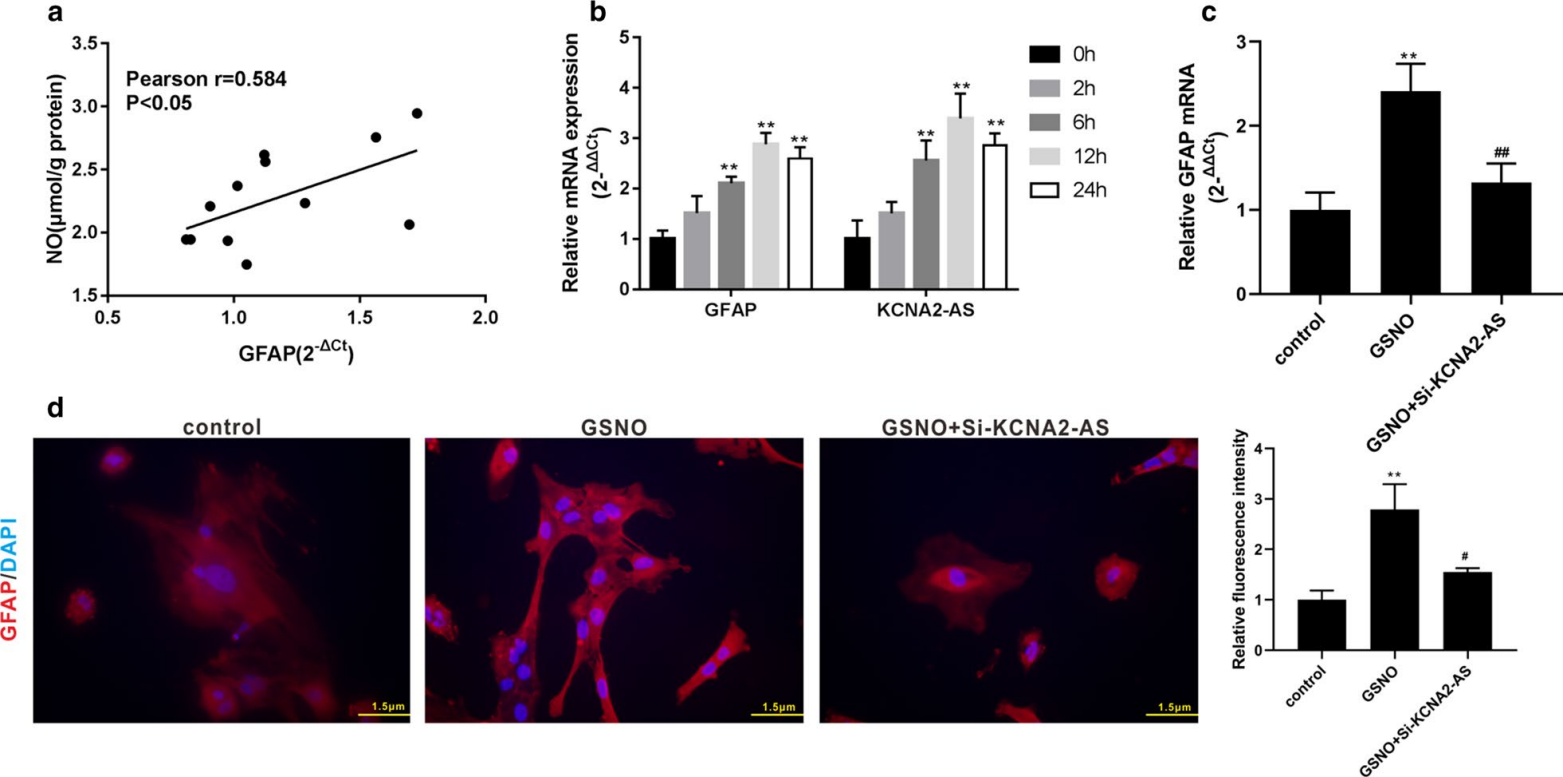
The suppression of KCNA2-AS inhibits the activation of NO to astrocytes. **a** As described previously, the correlation between NO content and GFAP expression in spinal cord tissues previously tested in vivo was analyzed. **b** Primary rat spinal astrocytes were treated with s-nitrosylglutathione (GSNO, NO donor) for different time periods (0, 2, 6, 12, and 24 h). The GFAP and KCNA2-AS expression were detected by qRT-PCR. **c**, **d** Primary rat spinal astrocytes were transfected with or without si-KCNA2-AS, and treated with GSNO for 24 h. The control group was untreated cells. The GFAP expression was detected by qRT-PCR (**c**). Immunofluorescence staining was performed by using GFAP antibody and DAPI. GFAP, red staining; DAPI / nucleus, blue staining (**d**). **P < 0.01 vs. 0 h group or control group; ^#^*P* < 0.05 and ^##^*P* < 0.01 vs. GSNO group

To explore whether KCNA2-AS was involved in spinal astrocyte activation and the further mechanism, we discovered that the content of NO was positively correlated with the expression of GFAP in the spinal cord of PHN rats (Fig. 3a). Therefore, we used NO to stimulate primary rat spinal cord astrocytes in vitro to study the possible mechanism of KCNA2-AS involved in spinal astrocyte activation. Primary rat spinal astrocytes were treated with s-nitrosylglutathione (GSNO; NO donor) for different time periods (0, 2, 6, 12 and 24 h), and the expression of GFAP mRNA and KCNA2-AS was measured. The results showed that the levels of GFAP mRNA and KCNA2-AS were gradually increased with time until 12 h (Fig. 3b). We treated primary rat spinal astrocytes with GSNO for 24 h, and the results showed that the level of GFAP mRNA was elevated (Fig. 3c). However, using si-KCNA2-AS to knock down the expression of KCNA2-AS in cells reversed the effect of GSNO on GFAP (Fig. 3c). Meanwhile, we used the GFAP antibody for immunofluorescence staining, and the results showed that GSNO increased the fluorescence intensity of GFAP, while si-KCNA2-AS reversed this result (Fig. 3d).

### KCNA2-AS binds to pSTAT3 and regulates its activation

**Fig. 4:**
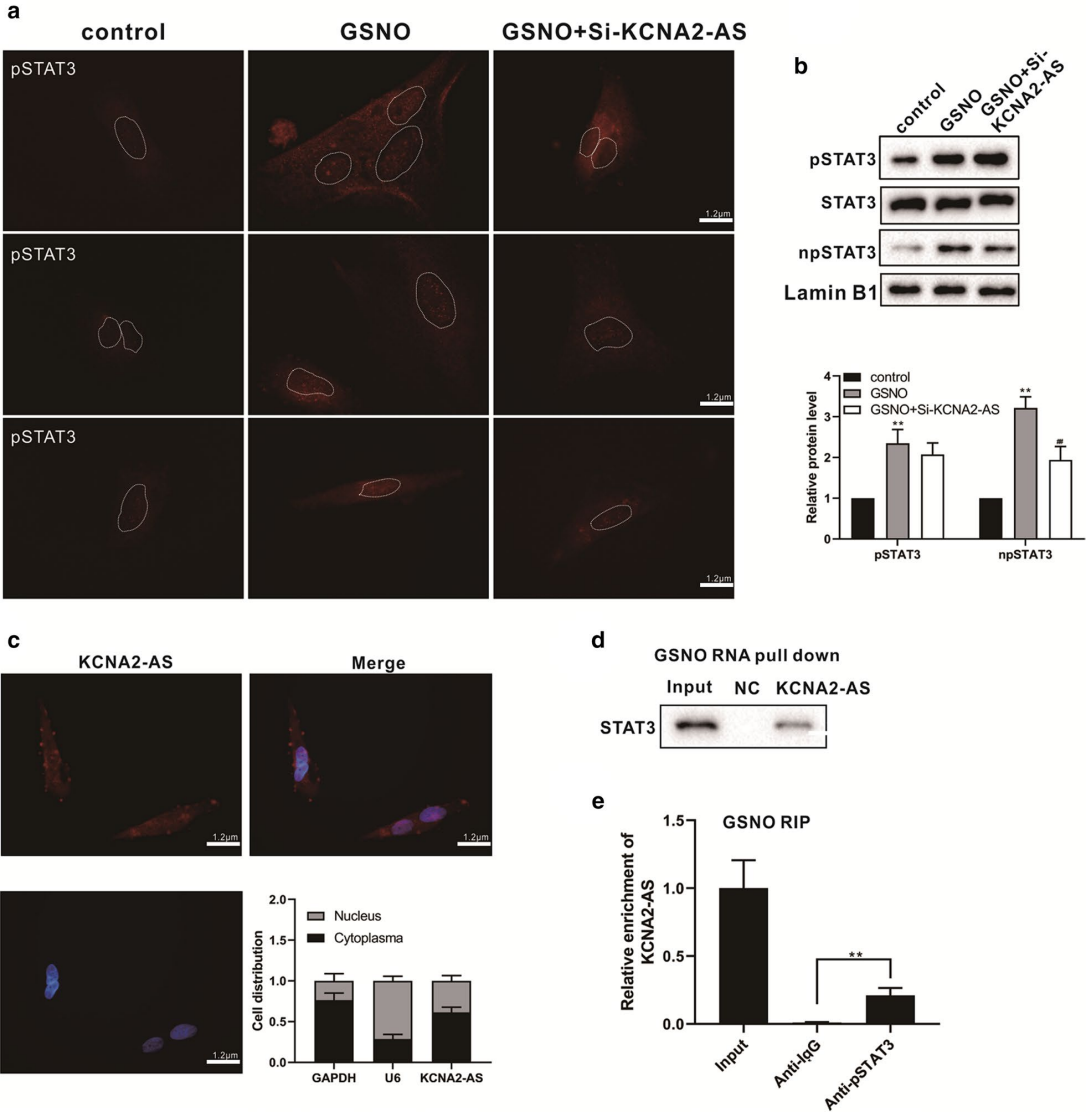
KCNA2-AS binds to pSTAT3 and regulates its activation. Primary rat spinal astrocytes were transfected with or without si-KCNA2-AS, and treated with GSNO for 24 h. The control group was untreated cells. **a** Immunofluorescence staining was performed with the pSTAT3 antibody. pSTAT3, red staining. **b** The nuclear or total STAT3 and pSTAT3 protein levels were detected by Western Blot. **P < 0.01 vs. control group; ^#^*P* < 0.05 vs. GSNO group. **c** RNA fluorescence in situ hybridization was performed. KCNA2-AS, red staining; DAPI/nucleus, blue staining. The nuclear or cytoplasm of KCNA2-AS levels were detected by qRT-PCR. **d**–**e** Primary rat spinal astrocytes were treated with GSNO for 24 h. The interaction between KCNA2-AS and pSTAT3 was assessed using RNA pull-down assay (**d**) and RIP assay (**e**). **P < 0.01 vs. Anti-IgG group

As previously mentioned, STAT3 phosphorylation (pSTAT3) promotes the activation of astrocytes (Ge 2018; Wang 2018), and we wanted to explore whether KCNA2-AS could regulate the activation of astrocytes through pSTAT3. Primary rat spinal astrocytes were transfected with or without si-KCNA2-AS, and treated with GSNO for 24 h. We used the pSTAT3 antibody for immunofluorescence staining, and the results showed that the fluorescence intensity of pSTAT3 was increased in the nucleus after GSNO treatment of cells (Fig. 4a). However, the fluorescence intensity of pSTAT3 was decreased in the nucleus after the transfection of si-KCNA2-AS (Fig. 4a). Meanwhile, we also detected the nuclear or total STAT3 and pSTAT3 protein levels. The results showed that the total STAT3 protein was not significantly different after treatment with GSNO, but the nuclear and total pSTAT3 protein levels of cells were significantly elevated (Fig. 4b). However, the level of nuclear pSTAT3 protein was significantly reduced after transfection with si-KCNA2-AS, but the level of total pSTAT3 protein was not significantly different (Fig. 4b).

We examined the distribution of KCNA2-AS in the nucleus and cytoplasm of primary rat spinal astrocytes by FISH or qRT-PCR, and we found that KCNA2-AS is distributed in both nucleus and cytoplasm (Fig. 4c). In order to detect whether KCNA2-AS binds to pSTAT3, RNA-pull down and RIP analyses were performed in GSNO-induced primary rat spinal astrocytes. The RNA pull-down results showed that a higher protein expression level of pSTAT3 in KCNA2-AS pulled down pellets (Fig. 4d), and the RIP results showed that KCNA2-AS was observably enriched in pSTAT3 precipitation (Fig. 4e). These results suggested that KCNA2-AS can bind to pSTAT3, and the reduction of KCNA2-AS inhibited the translocation of pSTAT3 to the nucleus in GSNO-induced primary rat spinal astrocytes.

### Knockdown of KCNA2-AS in the spinal cord of PHN rats alleviates neuropathic pain

**Fig. 5:**
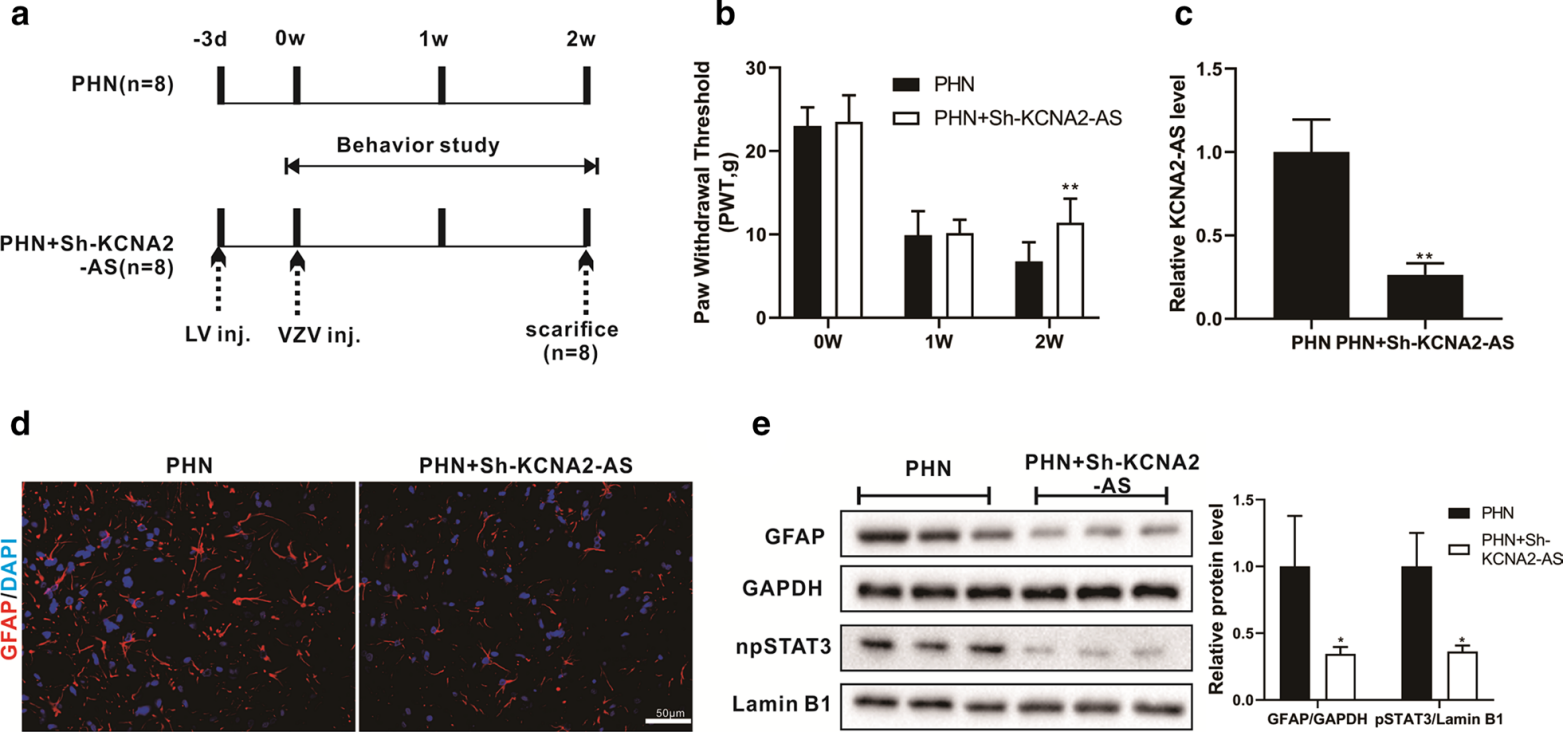
Knockdown of KCNA2-AS in the spinal cord of PHN rats alleviates neuropathic pain. Varicella zoster virus-infected CV-1 cells (monkey kidney cell line), and 5 × 10^6^ cells were injected into rats to construct a PHN rat model, and 3 days before, the rats were intrathecal injected (PHN + si-KCNA2-AS group) or no injection (PHN group) of LV-si-KCNA2-AS. The rats were sacrificed after 2 weeks. **a** Schematic diagram of rat treatment. **b** Mechanical pain was detected by the PWT method before or 1 and 2 weeks after injection in rats. **c** The levels of KCNA2-AS were detected in spinal cord tissue by qRT-PCR. **d** Immunofluorescence staining was performed on the sections of rat spinal cord. **e** The levels of pSTAT3 and GFAP protein were detected in spinal cord tissue by Western blot. *P < 0.05 and **P < 0.01 vs. PHN group

To further validate our results, we injected LV-sh-KCNA2-AS into the PHN rat model to knock down KCNA2-AS, and the rats were sacrificed after 2 weeks (Fig. 5a). Mechanical pain was detected by the PWT method after LV-sh-KCNA2-AS injection for 0 w, 1 w and 2 w. The results showed that mechanical pain was observably reduced in PHN model rats after LV-si-KCNA2-AS injection for two weeks (Fig. 5b). Moreover, 2 weeks after injection of LV-si-KCNA2-AS in PHN rats, KCNA2-AS was significantly reduced in spinal cord tissue (Fig. 5c). Immunofluorescence staining of rat spinal cord showed that the fluorescence intensity of GFAP was decreased after LV-si-KCNA2-AS injection (Fig. 5d). Meanwhile, the levels of pSTAT3 and GFAP protein were significantly reduced (Fig. 5e).

## Discussion

PHN is defined as pain that persists for at least 90 days after herpes zoster, and usually manifests as allodynia and hyperalgesia (Saguil 2017). Meanwhile, PHN is often debilitating and affecting the patient's physical function, mental health, and quality of life (Pickering and Leplege 2011). Nevertheless, the pharmacological treatment of PHN includes some tricyclic antidepressants, topical analgesics, and alpha-2 delta ligands that are either less effective or have more side effects (Hadley 2016). Therefore, it is necessary to explore the pathogenesis of PHN and find new treatment strategies for PHN. LncRNAs have been shown to play a crucial role in the development of neurons and the occurrence and progression of neuropathic pain (Li 2019; Wu et al. 2019; Qureshi and Mehler 2013). However, the role of lncRNAs in PHN has been rarely reported. We found that KCNA2-AS was highly expressed in the spinal cord tissue of PHN model rats, and knockdown of KCNA2-AS can alleviate mechanical allodynia. Besides, NO expression was also significantly increased in the spinal cord tissue of PHN model rats. This suggests that KCNA2-AS is closely related to NO expression and may be a potential target for the treatment of PHN.

The study found that the spinal dorsal horn and anterior cingulate gyrus cortex of PHN patients are involved in the regulation of pain information, and the related nuclei or brain regions exhibit excessive metabolism and activation (Zhang 2014). This suggests that the spinal dorsal horn may play a crucial role in PHN pathology. A study demonstrated that 9 months after spinal cord injury in rats, the markers of astrocyte activation GFAP is still significantly up-regulated, while the response of other cell types, such as microglia, is reduced (Gwak 2012). Specific activation of astrocytes in the spinal cord of rats or mice can cause mechanical allodynia in animals (Nam 2016; Jiang 2016). Furthermore, inhibition of spinal astrocyte activation can alleviate neuropathic pain (Ji et al. 2019). These results demonstrate that activation of spinal astrocytes is an essential factor in the occurrence and maintenance of neuropathic pain. Similarly, spinal astrocytes were obviously activated in the PHN model, but specific inhibition of their activation could obviously relieve mechanical pain (Zhang 2011; Zhang et al. 2009). Our study found that astrocyte activation marker GFAP was positively correlated with KCNA2-AS expression in the spinal cord of PHZ model rats. In addition, the expression of KCNA2-AS and GFAP were significantly elevated in activated primary spinal astrocytes, while knockdown of KCNA2-AS expression could significantly reduce GFAP levels in NO-induced primary rat spinal astrocytes. Similar to our findings, Zhang GH et al. also found that spinal astrocyte activation in PHN rat models, and inhibition of spinal astrocyte activation can alleviate mechanical allodynia and spinal central sensitization in rats (Zhang 2011). These results indicate that knockdown of KCNA2-AS in the spinal cord of PHN model rats can alleviate the neuropathic pain of rats by inhibiting NO-induced astrocyte activation.

Our study showed that pSTAT3 was increased after activation of primary spinal astrocytes. The STAT3 signaling pathway has been shown to be involved in the activation of astrocytes in neuropathic pain, and spinal STAT3 is phosphorylated and the STAT3 signaling pathway is activated in the neuropathic pain model (Ge 2018; Wang 2018). Moreover, inhibition of spinal pSTAT3 and STAT3 signaling pathway reduces pain in the neuropathic model (Wang 2014). Furthermore, some researches demonstrated that lncRNA could bond to pSTAT3 and affect the stability of pSTAT3 protein to achieve its regulatory function, such as lncRNA MEG3 (Zhang and Gao 2019).Our study found that KCNA2-AS bound to pSTAT3, and the reduction of KCNA2-AS inhibited the translocation of pSTAT3 to the nucleus in activated primary rat spinal astrocytes. These results indicate that KCNA2-AS can regulate the activation of spinal astrocytes by regulating the cytoplasmic/nuclear translocation of pSTAT3, thereby affecting PHN.

In summary, our study found that KCNA2-AS is highly expressed in the spinal cord tissue of PHN model rats, and knockdown of KCNA2-AS can alleviate the mechanical allodynia of PHN model rats. KCNA2-AS regulates PHN partly by combining with pSTAT3 to regulate cytoplasmic/nuclear translocation of pSTAT3, and then modulate NO-induced astrocyte activation, which affects the progression of PHN. This provides a new strategy for treating PHN.

## Data Availability

All data generated or analyzed during this study are included in this published article.
